# Adaptive and neutral markers both show continent‐wide population structure of mountain pine beetle (*Dendroctonus ponderosae*)

**DOI:** 10.1002/ece3.2367

**Published:** 2016-08-08

**Authors:** Philip D. Batista, Jasmine K. Janes, Celia K. Boone, Brent W. Murray, Felix A. H. Sperling

**Affiliations:** ^1^ Department of Biological SciencesUniversity of AlbertaEdmontonAlbertaT6G2E9Canada; ^2^ School of Environmental and Rural ScienceUniversity of New EnglandArmidaleNew South Wales2351Australia; ^3^ Biological Control and Spatial Ecology Lab (LUBIES)Université Libre de BruxellesBrusselsBelgium; ^4^ Natural Resources and Environmental Studies InstituteUniversity of Northern British ColumbiaPrince GeorgeBritish ColumbiaV2N 4Z9Canada

**Keywords:** Adaptive selection, *Dendroctonus ponderosae*, mountain pine beetle, population genetic structure

## Abstract

Assessments of population genetic structure and demographic history have traditionally been based on neutral markers while explicitly excluding adaptive markers. In this study, we compared the utility of putatively adaptive and neutral single‐nucleotide polymorphisms (SNPs) for inferring mountain pine beetle population structure across its geographic range. Both adaptive and neutral SNPs, and their combination, allowed range‐wide structure to be distinguished and delimited a population that has recently undergone range expansion across northern British Columbia and Alberta. Using an equal number of both adaptive and neutral SNPs revealed that adaptive SNPs resulted in a stronger correlation between sampled populations and inferred clustering. Our results suggest that adaptive SNPs should not be excluded prior to analysis from neutral SNPs as a combination of both marker sets resulted in better resolution of genetic differentiation between populations than either marker set alone. These results demonstrate the utility of adaptive loci for resolving population genetic structure in a nonmodel organism.

## Introduction

Neutral genetic markers have been considered essential to research on population structure (Luikart et al. 2003; Soto‐Cerda and Cloutier 2013), and numerous methods have been developed to identify and potentially remove outlier markers that may be under selection (Seeb et al. 2011). However, the practice of removing adaptive markers in order to determine population structure has recently been questioned because adaptive markers may contribute to the very structure that is being sought (Heylar et al. 2011). In some cases, adaptive single‐nucleotide polymorphism (SNP) markers may define management or conservation units more effectively than putatively neutral markers such as microsatellites (e.g., Russello et al. 2012; Milano et al. 2014; Moore et al. 2014). Adaptive markers may also help to identify populations experiencing different ecological conditions, such as temperature or salinity (Nosil et al. 2009; Heylar et al. 2011; Milano et al. 2014). Therefore, it should be useful to include markers under selection in surveys of population structure, to allow greater understanding of evolutionary processes and support more effective management of populations, especially in nonmodel organisms.

**Figure 1:**
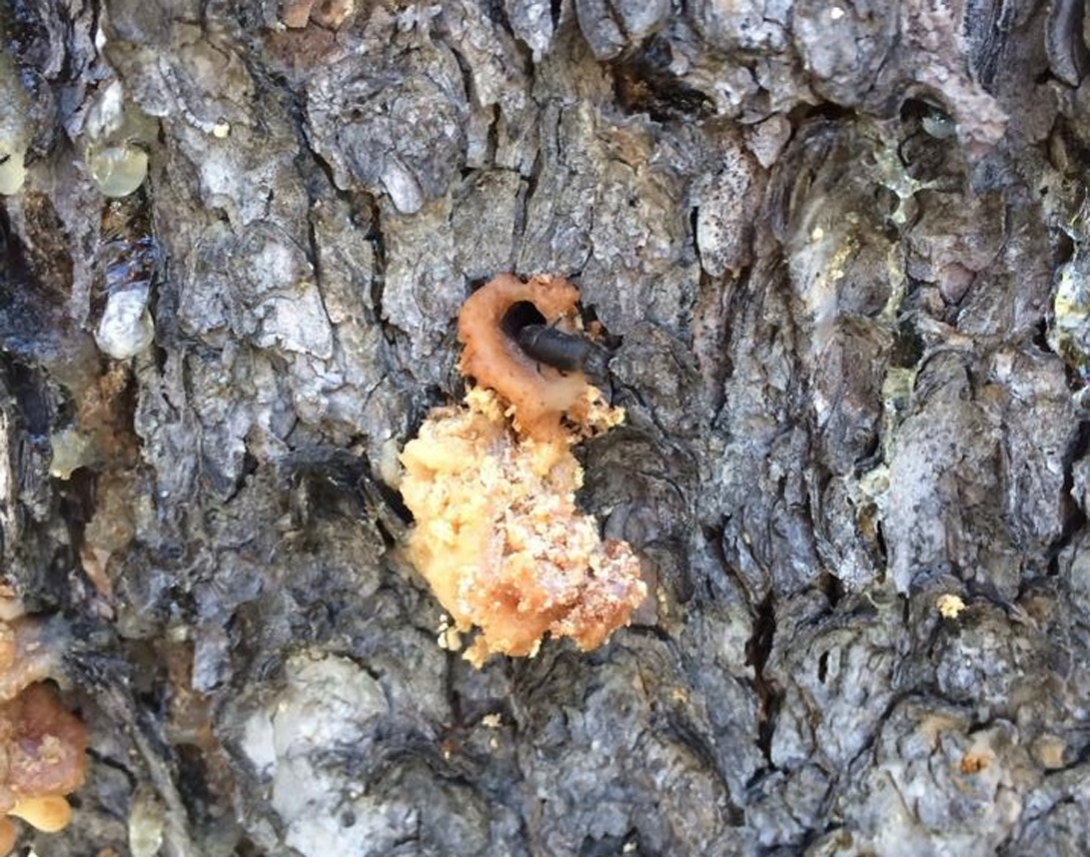
Mountain pine beetle found outside pitch tube of a lodgepole pine.

The mountain pine beetle (MPB), *Dendroctonus ponderosae* Hopkins (Coleoptera: Scolytinae), occurs throughout much of western North America as a native pest of mature pine forest (Safranyik et al. 1974). Until recently, its distribution extended from northern Mexico to northwestern British Columbia (BC), and from the Pacific coast to as far east as the Black Hills of South Dakota (Safranyik et al. 2010). Throughout its geographic range, MPB colonizes various pine hosts (Fig. 1), particularly ponderosa pine (*Pinus ponderosa* C. Lawson), western white pine (*Pinus monticola* Douglas *ex* D. Don), and lodgepole pine (*Pinus contorta* Dougl. *ex* Loud. var. latifolia; Safranyik et al. 1974; Taylor et al. 2006). In its endemic state, MPB only colonizes weakened or dying trees; however, during epidemic outbreaks, MPB is capable of killing healthy mature pines, making it one of the most destructive forest pests in western North America (Safranyik and Carroll 2006). Changing climate and forestry practices have now opened up previously unsuitable habitat in northern Canada to MPB (Carroll et al. 2003). In 2006, it became evident that an ongoing MPB outbreak and eastward range expansion could allow MPB to establish in the boreal forest of Canada (Safranyik and Carroll 2006), as central Alberta (AB) populations shift to jack pine (*Pinus banksiana*), a novel host (Safranyik et al. 2010; Cullingham et al. 2011).

Early studies of population structure in MPB used allozymes to characterize genetic variation, and in practice, most of these markers were considered neutral (Richardson et al. 1986; Volis et al. 2005; Beebee and Rowe 2008). Two studies reported low gene flow, with one identifying a north–south cline within Idaho (Stock and Guenther 1979), while the second found no support for divergence of populations across western USA that had previously been treated as a separate species, *D. monticolae* (Stock et al. 1984). In contrast, studies that partitioned beetle genetic variation by host tree showed local genetic differentiation among MPB populations in Colorado (Sturgeon and Mitton 1986). Langor and Spence (1991) found similar substructuring within Alberta, which they attributed to the differential survival of larvae within host trees rather than discrete host preferences among MPB genotypes.

More recent studies have used DNA to examine the population structure of MPB and have again mainly relied on putatively neutral loci. Mitochondrial DNA (mtDNA) sequences and amplified fragment length polymorphisms (AFLPs) showed isolation‐by‐distance (IBD) patterns around the US Great Basin (Mock et al. 2007) and in western Canada (Cullingham et al. 2011). These patterns of genetic divergence in western USA were supported by parallel patterns of hybrid male sterility (Bracewell et al. 2010). More recently, microsatellite loci revealed separation between southern and northern MPB populations in western Canada (James et al. 2011; Samarasekera et al. 2012), confirming the area around Tweedsmuir Provincial Park as the likely source of the recent northern outbreak in Canada (Aukema et al. 2006; Samarasekera et al. 2012). Janes et al. (2014) then surveyed variation in SNPs that were mainly selected for their potential functional role in MPB biology, confirming the north–south structuring of MPB populations in western Canada. This study suggested that multiple source populations contributed to the northern expansion and attributed the successful expansion to several outlier loci associated with cellular or metabolic functions.

Thus, with the exception of Janes et al. (2014), previous studies on MPB population structure have been implicitly based on neutral loci. However, Safranyik and Carroll (2006) have argued that neutral markers do not identify historical patterns in genetic structure in MPB, because contemporary processes such as population mixing and long‐distance dispersal would obscure any historical signature. Thus, MPB is ideal for exploring the limits of our current understanding of neutral markers, and provides an opportunity to ask whether markers under selection are better for detecting population divergence. The aim of this study was to test whether putatively adaptive markers have the same information content as neutral markers for showing population structure. We compare genetic structure patterns in MPB across its historical and expanded geographic range using (1) SNPs previously identified as being under selection (Janes et al. 2014); (2) probable neutral loci; and (3) a combined data set to determine if adaptive and neutral loci perform better when pooled. Further, identification of signals of selection on these loci within the different regions of the MPB range was carried out using an outlier detection test.

## Methods

### Sample collection and SNP genotyping

Mountain pine beetle specimens were collected from 62 sites across Canada and the USA (Table S1), with emphasis on Canadian populations. Genomic DNA was extracted from late instar larvae and adults using the DNeasy 96 Blood and Tissue Kit (Qiagen, Toronto, Ontario), following the manufacturer's instructions, or phenol–chloroform extraction as outlined in Samarasekera et al. (2012). Genomic DNA was quantified using a Qubit Fluorometer (Invitrogen, Burlington, Ontario). All samples were diluted to 20 ng/*μ*L. Genotyping was performed using the Sequenom iPLEX Gold assay (Sequenom 2008) at the Génome Québec and McGill University Innovation Centre (Janes et al. 2016). Selection of adaptive SNPs for this study was based on the panel of 1546 SNPs previously developed using markers with potentially important physiological functions (Janes et al. 2014).

Based on their identification as outliers by Janes et al. (2014) using BayeScan (Foll and Gaggiotti 2008) and Lositan (Antao et al. 2008), we grouped the SNPs into two sets – adaptive and neutral. We then developed a Sequenom panel of autosomal SNPs that comprised both putatively neutral and adaptive SNPs. The adaptive set was comprised of SNPs repeatedly detected as outliers, under positive selection, with high significance. The neutral set consisted of SNPs not detected as outliers, or weakly detected by one program. All samples and SNPs in this study were filtered prior to analysis to exclude low‐quality SNPs that had an average call rate <80% (i.e., SNPs unsuccessfully genotyped) as per Janes et al. (2016), and samples with >10% of SNPs missing information (i.e., undetermined bases). The final data set comprised 1115 individuals and 92 SNPs, of which 36 SNPs were assigned as adaptive and 56 as neutral.

### Genetic structure

Population genetic structure was determined using three data sets: (1) all available SNPs, as “combined”; (2) putatively adaptive SNPs; and (3) putatively neutral SNPs. Pairwise *F*_ST_ measures for all 62 populations were calculated to evaluate genetic differentiation among MPB populations and tested with 10,000 permutations using Arlequin 3.5.2 (Excoffier and Lischer 2010). Discriminant analysis of principal components (DAPC) implemented in the R (R Core Team, 2014) package adegenet (Jombart 2008; Jombart and Ahmed 2011) was used in a multivariate analysis to identify the number of clusters (*K*) of genetically related individuals. The number of retained principal components (PC) was determined using the *optim.a.score* function for each data set. We retained seven PCs for the neutral SNPs, 24 for the adaptive SNPs, and 14 for the combined panel of SNPs. The Bayesian information criterion was used to select the optimal number of *K* clusters.

A complementary method used model‐based Bayesian clustering implemented in STRUCTURE v.2.3.4 (Pritchard et al. 2000; Falush et al. 2003). STRUCTURE was run for *K* 1–10 using the following parameters: admixture ancestry model and correlated allele frequencies, 10,000 burn‐in, 100,000 MCMC repetitions, and 10 iterations of each *K*. The Delta *K* method of Evanno et al. (2005) implemented in STRUCTURE HARVESTER (Earl and von Holdt 2012) identified the optimal *K*. Alignment of repetitions for the best *K* used CLUMPP v1.1.2 (Jakobsson and Rosenberg 2007). A range of *K*‐values were selected to compare the population assignment of individuals to clusters between STRUCTURE and DAPC. As sampling density differed between the US and Canada, three data sets containing an equal number of randomly selected Canadian populations to US populations were created for each of the adaptive, neutral, and combined sets. A graphical representation of the average probability assignment of each population to a cluster was mapped using ArcGIS 10.2.2 (ESRI 2014).

### Data set comparisons

Comparisons of STRUCTURE results from each of the data sets (combined, adaptive, and neutral) were conducted with ObStruct (Gayevskiy et al. 2014). ObStuct quantifies the contribution of each predefined sample population (i.e., 62 sampling locations) to its inferred population assignment using the ancestry proportions generated by STRUCTURE from each marker type, which are the individual probability assignment to a subpopulation (inferred *K* cluster). Using this data, ObStruct generates a multiple *R* ^2^ statistic that is used to explain how much of the variability is explained by the sampling locations. This correlation, presented as a single *R* ^2^ statistic, is interpreted such that a low *R* ^2^ indicates no significant structure between populations and a high *R* ^2^ indicates strong population structure.

The unequal number of SNPs in adaptive versus neutral sets presented a problem as a larger number of markers should provide a more defined assessment of population structure regardless of their identity. We removed the bias in favor of the larger cohort of neutral SNPs by using three randomized subsets of the neutral set that were equal in marker number to the adaptive set. These subsets were based on randomized sampling without replacement of the larger data set, prior to comparing the *R* ^2^ between the data sets.

### Outlier detection

Outlier detection analyses remain contentious because different selection tests make different assumptions about the demographic and genetic structure of a population (reviewed in Nielsen 2005; Vitti et al. 2013). For example, natural selection may indirectly influence neutral variation through linked adaptive loci, a phenomenon referred to as genetic hitchhiking. With this in mind, we determined whether the same SNPs were identified as putative outliers across each of the population groupings. Detection of outliers for different regional groups used a Bayesian approach implemented in BayeScan v.2.1 (Foll and Gaggiotti 2008). This method applies linear regression to decompose *F*_ST_ coefficients into population‐ and locus‐specific components and estimates the posterior probability of a locus showing significant deviation from Hardy–Weinberg proportions by a reversible‐jump MCMC approach (Foll and Gaggiotti 2008). Each data set was run in triplicate, with a burn‐in of 50,000, a thinning interval of 10, and a sample size of 5000. Outlier plots were visualized in R (R Core Team, 2014).

## Results

### Genetic structure

Pairwise *F*_ST_ values for the 62 populations showed low values among regional groupings (Table S2). *F*_ST_ values between northern Canadian populations and southern Canada averaged 0.106, whereas average pairwise distances within these two regions were 0.019 and 0.026, respectively. South Dakota and Arizona had the largest differences from other regional groupings, ranging from 0.267 for US Rocky Mountain populations (Colorado, Wyoming and Nevada) to an average of 0.376 in comparisons with northern Canadian populations.

**Figure 2:**
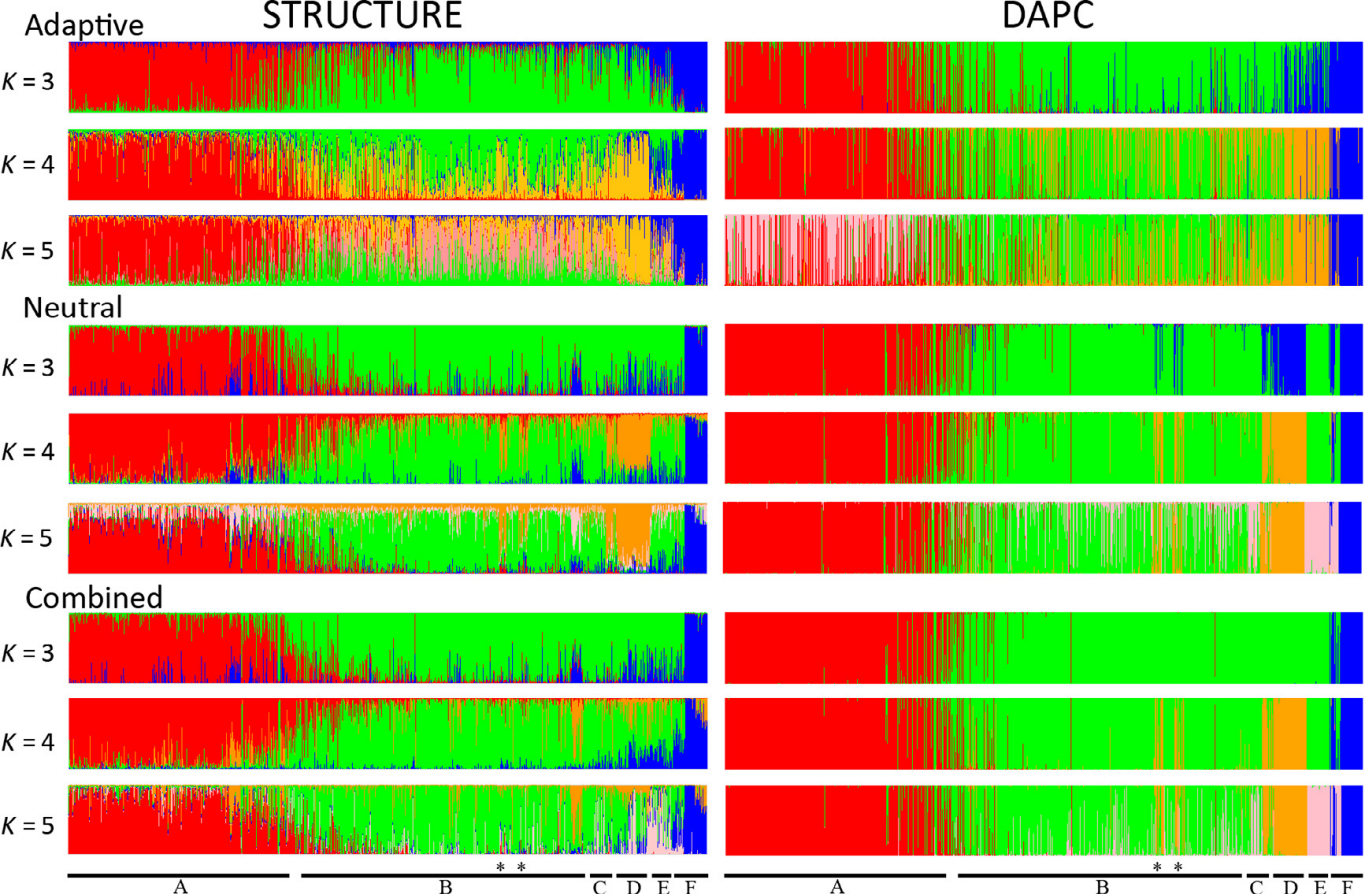
STRUCTURE and discriminant analysis of principal components plots of North American *Dendroctonus ponderosae* populations for *K *=* *3–5 using putatively adaptive loci, neutral loci, and both adaptive and neutral loci combined. Regions underlined below represent: (A) northern Canada; (B) southern Canada; (C) Idaho, Montana, and Washington; (D) Oregon, California, and Nevada; (E) Utah and Wyoming; and (F) Arizona and South Dakota. Stars indicate Whistler and Manning Park.

In each data set (combined, neutral and adaptive), STRUCTURE analysis showed *K *=* *2 as optimal, separating northern Canadian populations from the southern Canadian and US populations. All randomized data sets reflected this optimal *K *=* *2. STRUCTURE analysis at *K *=* *3 for each of the data sets also distinguished the populations from South Dakota, Arizona, and part of Colorado (Fig. 2: blue), while *K *=* *4 showed clustering of western USA from the remaining US populations in both the neutral and adaptive data sets (Fig. 2: orange and Fig. S1). At *K *=* *4 in the neutral and combined data sets, beetles from Manning Park and Whistler in British Columbia clustered with MPB populations along the west coast USA (Oregon, California, and Nevada: orange). For adaptive SNPs, population groupings are less distinct in each plot, and although some genetic differentiation of west coast US populations can be seen, adaptive SNPs did not identify the Manning Park and Whistler populations as a separate cluster. Further analysis (*K *=* *5) did not differentiate US Rocky Mountain populations (Colorado, Wyoming, and Nevada: pink) from the remaining US populations.

**Figure 3:**
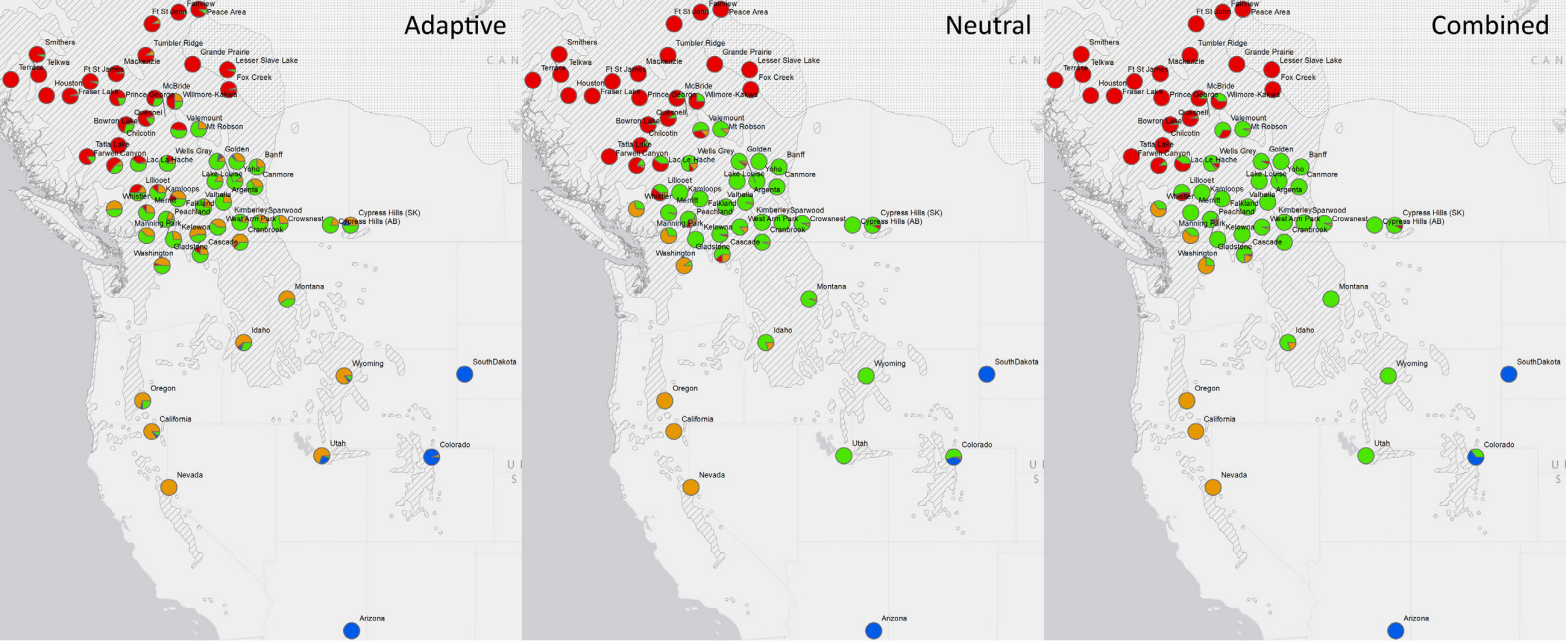
Cluster assignments from discriminant analysis of principal components for three sets of single‐nucleotide polymorphisms at *K* = 4. The colors in each pie chart correspond to the assignment probability, with the four general regions identified as follows: Red* *=* *northern Canada; Green* *=* *southern Canada and northern USA; Orange* *=* *west coast populations; Blue* *=* *outlying southern populations. Mountain pine beetle sampling is overlaid on lodgepole (hatched area) and jack pine (dotted area) distributions.

Discriminant analysis of principal components analyses largely paralleled the STRUCTURE results, including initial differentiation of northern Canada at *K *=* *2 for each data set. Unlike the STRUCTURE results, the optimum *K* value for DAPC was *K *=* *8, resulting in several broadly overlapping clusters that were difficult to interpret (Fig. S2, Tables S3–S5). However, the bar graph plots for DAPC for *K *=* *3 to *K *=* *5 showed substantial similarity to the corresponding STRUCTURE plots for each of the data sets (Fig. 2). As with the STRUCTURE results, the adaptive SNPs showed less distinct geographic groupings than either the neutral or combined SNP sets, including poor genetic differentiation of western US populations or the clustering of the Manning Park and Whistler populations (Fig. 3). For *K* = 3, unlike the STRUCTURE results for putatively neutral loci, west coast US populations grouped with South Dakota and Arizona.

### Data set comparisons

Ancestry profiles from STRUCTURE for *K *=* *4 from each data set were compared using ObStruct. This analysis revealed that the randomized adaptive SNPs had a larger *R* ^2^ statistic (*R* ^2^ **=* *0.81), indicating that the variability of the data was best explained by sampling locations, than each of the three randomized subsets of neutral SNPs (*R* ^2^ **=* *0.76–0.77). Assignments based on the full set of neutral SNPs resulted in an increase in the *R* ^2^ statistic (*R* ^2^ **=* *0.83), as did the combined set containing both neutral and adaptive SNPs (*R* ^2^ **=* *0.85).

### Outlier detection

Using the combined data set, BayeScan was used to identify loci that might show differing degrees of selection within the different regional groupings of MPB populations. The clustering assignments from STRUCTURE and DAPC were used to partition the different populations for outlier detection. No outliers were detected within the US clusters (Arizona and South Dakota; Wyoming, Utah, and Colorado; and west coast USA), or within northern Canadian populations.

Examination of southern Canadian/northern US populations identified 12 outlier loci, two of which were noncoding. The SNPs in coding regions with a putative protein function included the following: a gene encoding a ribonuclease CAF1 (locus 0103), ribosomal protein L9 (locus 0147), vacuolar protein sorting‐associated protein 51 (Vps51) (locus 0369), proliferation‐associated protein 2G4 (locus 0821), ABC transporter G family member (locus 0950), major facilitator superfamily (MFS) protein (locus 0981), family with sequence similarity 60 member A (FAM60A) protein (locus 1045), Beige and Chediak‐Higashi (BEACH) domain‐containing protein (locus 1069), and a cold shock‐containing protein (locus 1529).

## Discussion

This study documents the first explicit use of adaptive SNPs to assess the range‐wide population structure of an invasive insect pest. Adaptive SNPs detected in MPB populations may be important for identifying local variation and selection processes in regions where ecological adaptation to different conditions is leading to increased fitness (Nosil et al. 2009; Matala et al. 2014). We compare putatively adaptive SNPs, which are typically excluded prior to any population genetic analysis, to putatively neutral SNPs to determine whether the outlier SNPs also contribute to MPB population structure.

### Patterns of population genetic structure

Our results from DAPC and STRUCTURE analyses of adaptive SNPs were consistent with previous work that examined MPB population structure in western Canada. Differentiation of northern Canadian populations from more southern populations was seen in every data set. This subdivision of northern Canadian populations, which have recently undergone a range expansion, was identified previously using microsatellites (James et al. 2011; Samarasekera et al. 2012), SNPs (Janes et al. 2014) and mitochondrial DNA sequence (Cullingham et al. 2012).

For range‐wide structure of MPB, Mock et al. (2007) showed that Arizona populations were most genetically similar to populations from Utah. In this study, DAPC results showed that Arizona and South Dakota were the most genetically similar, while Utah and Wyoming clustered separately from Arizona and South Dakota (Fig. 3). STRUCTURE analyses indicated gene flow occurring from Arizona into Utah with all data sets, in addition to the relationship between Arizona and South Dakota. This discrepancy with Mock et al. (2007) may be due to the increased level of sampling in our study or an artifact of DAPC analyses that appear to be more sensitive than those of STRUCTURE. One potential explanation for the genetic relatedness of MPB from South Dakota and Arizona may be northward transport of infected wood; however, this hypothesis needs to be tested with further sampling.

The combined data set identified substructuring of Whistler with Manning Park, suggesting that these populations originated from west coast US populations, with subsequent differentiation. Samarasekera et al. (2012) and Janes et al. (2014) found that Whistler was genetically different from other Canadian populations, but each provided slightly different explanations for the pattern. Janes et al. (2014) attributed the pattern to continued low immigration numbers from northern US populations into Whistler, coupled with slower dispersal from the Whistler area, east in southern Canada, as a result of low‐density forest (Janes et al. 2014). Alternatively, Samarasekera et al. (2012) explained the pattern as the result of lower immigration into the Whistler area due to the prevalent west to east winds. Our DAPC analyses showed that a portion of the individuals from Whistler and Manning Park in southern Canada, and Washington from northern USA, were assigned to the genetic cluster containing individuals from Oregon, California, and Nevada. The incomplete assignment of all individuals suggests continued, low gene flow through migration among west coast US populations.

Clustering of individuals from southern Alberta and British Columbia with those of Washington, Idaho, and Montana shows a shared genetic history of populations along the distribution of lodgepole pine around the Great Basin Desert. These results support the isolation‐by‐distance pattern of gene flow that Mock et al. (2007) saw around the Great Basin Desert with gene flow occurring around the Great Basin and Mojave Deserts as a result of sporadic host pine distribution within these areas (Mock et al. 2007). Essentially, this pattern results in some of the most spatially proximal MPB populations in the USA displaying the highest genetic divergence between them. This basin appears to be the largest geographic barrier for MPB and may have led to reproductive isolation occurring between eastern and western US populations (Bracewell et al. 2010).

### Outlier detection

Candidate loci within different partitions of MPB populations based on DAPC clustering revealed few associated outliers. Previous work in detecting adaptive loci in Canadian populations by Janes et al. (2014) found SNPs belonging to three categories: ion transport, actin contraction, and sterol association. Using a subset of the SNPs of Janes et al. (2014) and some putatively neutral SNPs, our study detected few outliers within each subpopulation. Inclusion of adaptive loci in detection of outliers can bias which genes are identified as being under selection because it will first detect those genes that have extremely high *F*_ST_ values.

Within southern Canadian populations, outlier detection identified several SNPs associated with cell cycle and DNA and RNA processing proteins. Two cell‐cycle regulation proteins were identified, including a proliferation‐associated protein 2G4 (PA2G4) that is an RNA‐binding protein with a potential role in cell‐cycle control. The second is a FAM60A protein, a subunit of the SIN3A‐HDAC complex that acts as a regulator of gene expression by deacetylating histones (Muñoz et al. 2012).

The DNA and RNA processing proteins detected as outliers in southern Canadian populations included a cold shock domain‐containing protein that has numerous processes linked to RNA metabolism (Mihailovich et al. 2010). A second protein contained the N‐terminal domain of the ribosomal protein L9, a component of the large ribosomal subunit. A third protein was ribonuclease CAF1, which is associated with a CCR4‐NOT complex, an evolutionary conserved protein complex in eukaryotic cells that is essential for mRNA metabolism (reviewed in Denis and Chen 2003). Janes et al. (2014) previously identified this CCR4‐NOT associated protein as being under selection among southern Canadian populations. These outliers may indicate regional differences in spring development of MPB, where changes in enzymes involved in RNA metabolism have been reported (Bonnett et al. 2012).

Southern populations also had outlier proteins involved in cellular transport. These include a Vps51 subunit of the GARP complex associated with vesicular transport (Bonifacino and Hierro 2011). Another outlier protein identified belongs to the major facilitator superfamily (MFS), which includes a group of transporters involved in the transport a variety of substrates across the membrane. Selection was also inferred for an ABC transporter belonging to the G‐subfamily. Characterized in Drosophila, members of this subfamily are responsible for the transport pigment precursors into pigment cells (Mackenzie et al. 1999).

The lack of SNPs identified as being under positive selection in Arizona and South Dakota or Utah, Wyoming, and Colorado might be explained by the small sample sizes for these states, or genetic drift and selection processes acting differently between relatively recent Canadian populations and more historical US populations. In any case, although outlier detection methods are useful in identifying candidate genes under directional selection, actual functional characterization is required to demonstrate the role that these adaptive traits may have in different MPB populations.

### Performance of adaptive and neutral data sets

Adaptive and neutral markers showed largely concordant results for the overall population structure of MPB (Fig. 2). However, using ObStruct to evaluate the relative structure of the data sets, differences were identified in performance between neutral and adaptive markers. Ancestry proportions for each of the neutral and adaptive data sets (membership of individuals from sampled population to an inferred cluster) from STRUCTURE for *K *=* *4 were used for analysis. The *R* ^2^ statistic from ObStruct analysis between the randomized neutral loci (*R* ^2^ **=* *0.76–77) and adaptive loci (*R* ^2^ **=* *0.81) showed that sampling locations for each individual had a stronger correlation with their inferred population cluster using the adaptive markers. According to Gayevskiy et al. (2014), the lower *R* ^2^ value from our randomized neutral data set may suggest that these putatively neutral SNPs are more sensitive to migration and admixture. An increase in the *R* ^2^ value is seen when all neutral markers are combined (*R* ^2^ **=* *0.83) in addition to combining both adaptive and neutral markers (*R* ^2^ **=* *0.85), indicating that increasing the number of markers for STRUCTURE analysis leads to more differentiation between inferred populations. Taken together, these results show that the removal of adaptive loci prior to analyses is not always advisable as these loci may be helpful in identifying differentiation between populations when combined with neutral markers, and can potentially identify fine‐scale structure to define management units.

## Conclusion

This study shows that outlier loci can be highly informative for determining the population structure of MPB across its entire range. This challenges the traditional population genetic paradigm of using only neutral markers. Moreover, with the increasing availability of SNP data, this demonstrates the value of including adaptive loci, whether alone or in conjunction with traditionally neutral loci, for population genetic studies.

## Conflict of Interest

None declared.

## Data accessibility

All data used in this article will be available in Dryad Digital Repository (http://dx.doi.org/10.5061/dryad.540bt).

[Correction added on 06 September 2016, Data Accessibility Section is now added in this version.]

## Supporting information


**Table S1.** Site locations, coordinates, and collection years of sampled mountain pine beetle.
**Table S3.** Cluster assignment of individuals from DAPC analysis, all SNPs.
**Table S4.** Cluster assignment of individuals from DAPC analysis, neutral markers.
**Table S5.** Cluster assignment of individuals from DAPC analysis, adaptive markers.
**Figure S1.** Cluster assignments from STRUCTURE for three sets of SNPs for *K* = 4. The four general regions identified from the combined SNPs include northern Canada (red); southern Canada and Idaho, Montana, and Washington (green); Oregon, California, and Nevada (orange); Utah and Wyoming Colorado, Arizona, and South Dakota (blue).
**Figure S2.** DAPC clustering of each marker set for *K* = 8.

**Table S2.** Pairwise values of *F*_ST_ calculated between mountain pine beetle populations.

**Data S1.** SNP list.
