# Supplementary material for: Adaptive and neutral markers both show continent‐wide population structure of mountain pine beetle (Dendroctonus ponderosae)
Source: Ecol Evol. 2016 Aug 8;6(17):6292–300. doi: 10.1002/ece3.2367 (PMC5016649; doi:10.1002/ece3.2367)
Supplement: Supplementary file 1 — Table S1. Site locations, coordinates, and collection years of sampled mountain pine beetle. Table S3. Cluster assignment of individuals from DAPC analysis, all SNPs. Table S4. Cluster assignment of individuals from DAPC analysis, neutral markers. Table S5. Cluster assignment of individuals from DAPC analysis, adaptive markers. Figure S1. Cluster assignments from STRUCTURE for three sets of SNPs for K = 4. The four general regions identified from the combined SNPs include northern Canada (red); southern Canada and Idaho, Montana, and Washington (green); Oregon, California, and Nevada (orange); Utah and Wyoming Colorado, Arizona, and South Dakota (blue). Figure S2. DAPC clustering of each marker set for K = 8. [file ECE3-6-6292-s001.docx]

Supplementary Table and Figures

Table S1. Site locations, coordinates and collection years of sampled mountain pine beetle.

| Population | n | Longitude | Latitude | Collection Date |
| --- | --- | --- | --- | --- |
| Argenta | 20 | 50.1578 | -116.9173 | 2005/2006 |
| Arizona | 20 | 32.6620 | -109.8719 | 2011/2012 |
| Banff | 20 | 51.1779 | -115.5588 | 2005/2006 |
| Bowron Lake | 20 | 53.2488 | -121.4172 | 2005/2006 |
| California | 20 | 41.6806 | -120.8672 | 2011/2012 |
| Canmore | 18 | 50.9323 | -115.3364 | 2007/2008 |
| Cascade | 20 | 49.0166 | -118.2004 | 2009/2010 |
| Chilcotin | 9 | 52.1520 | -122.5717 | 2007/2008 |
| Colorado | 20 | 40.6205 | -105.4618 | 2011/2012 |
| Cranbrook | 19 | 49.4086 | -115.6462 | 2005/2006 |
| Crowsnest | 18 | 49.6574 | -114.5525 | 2007/2008 |
| Cypress Hills (AB) | 11 | 49.5931 | -110.0363 | 2009/2010 |
| Cypress Hills (SK) | 12 | 49.5899 | -109.9352 | 2009/2010 |
| Fairview | 20 | 56.5994 | -119.386 | 2007/2008 |
| Falkland | 20 | 50.52 | -119.6018 | 2005/2006 |
| Farwell Canyon | 20 | 51.6665 | -122.9033 | 2005/2006 |
| Fox Creek | 20 | 54.4806 | -116.6348 | 2007/2008 |
| Fraser Lake | 20 | 53.9791 | -125.0690 | 2005/2006 |
| Fort St James | 20 | 54.4431 | -124.2542 | 2005/2006 |
| Fort St John | 11 | 56.2465 | -120.8476 | 2005/2006 |
| Gladstone | 20 | 49.2591 | -117.9275 | 2007/2008 |
| Golden | 19 | 51.0744 | -116.3816 | 2007/2008 |
| Grande Prairie | 20 | 54.9924 | -118.6135 | 2007/2008 |
| Houston | 11 | 53.994 | -126.6527 | 2005/2006 |
| Idaho | 20 | 45.2290 | -115.8085 | 2011/2012 |
| Kamloops | 20 | 50.4859 | -120.5319 | 2005/2006 |
| Kelowna | 11 | 49.9965 | -119.6693 | 2005/2006 |
| Kimberley | 20 | 49.6962 | -115.9601 | 2005/2006 |
| Kootenay/Yoho | 18 | 51.1229 | -116.2908 | 2007/2008 |
| Lac Le Hache | 20 | 51.7307 | -121.5984 | 2005/2006 |
| Lake Louise | 20 | 51.4172 | -116.1793 | 2005/2006 |
| Lesser Slave Lake | 19 | 54.8193 | -116.6917 | 2009/2010 |
| Lillooet | 20 | 50.4566 | -121.635 | 2005/2006 |
| Mackenzie | 20 | 54.6963 | -122.821 | 2005/2006 |
| Manning Park | 16 | 49.2162 | -121.0697 | 2005/2006 |
| McBride | 20 | 53.3116 | -120.1266 | 2005/2006 |
| Merritt | 20 | 50.0352 | -120.6562 | 2005/2006 |
| Montana | 20 | 46.8915 | -113.4342 | 2011/2012 |
| Mt Robson | 20 | 52.8949 | -118.7348 | 2005/2006 |
| Nevada | 18 | 39.3437 | -119.9171 | 2011/2012 |
| Oregon | 20 | 42.9306 | -121.3639 | 2011/2012 |
| Peace Area | 20 | 56.7073 | -118.2762 | 2009/2010 |
| Peachland | 20 | 49.7971 | -120.057 | 2009/2010 |
| Prince George | 5 | 53.9170 | -122.7496 | 2005/2006 |
| Quesnel | 17 | 53.037 | -122.2741 | 2005/2006 |
| Smithers | 17 | 54.7823 | -127.1685 | 2009/2010 |
| South Dakota | 20 | 43.9835 | -103.6402 | 2009/2010 |
| Sparwood | 20 | 49.68 | -114.911 | 2007/2008 |
| Tatla Lake | 16 | 51.9715 | -124.413 | 2005/2006 |
| Telkwa | 20 | 54.6674 | -127.0887 | 2005/2006 |
| Terrace | 14 | 54.8365 | -122.508 | 2009/2010 |
| Tumbler Ridge | 20 | 55.2738 | -121.1913 | 2007/2008 |
| Utah | 20 | 40.6724 | -111.5081 | 2011/2012 |
| Valemount | 15 | 52.8532 | -119.3816 | 2007/2008 |
| Valhalla | 13 | 49.7503 | -117.5181 | 2005/2006 |
| Washington | 20 | 48.1295 | -120.2981 | 2011/2012 |
| Wells Grey | 20 | 51.7411 | -120.012 | 2005/2006 |
| West Arm Park | 20 | 49.5244 | -117.2324 | 2005/2006 |
| Whistler | 17 | 50.1678 | -122.9251 | 2005/2006 |
| Wilmore-Kakwa | 16 | 53.8036 | -119.6004 | 2007/2008 |
| Wyoming | 20 | 43.9239 | -110.2876 | 2011/2012 |
| Yoho | 10 | 51.1229 | -116.2908 | 2007/2008 |

Table S3: Cluster assignment of individuals from DAPC analysis, all SNPs.

| Region | Clusters | | 1 | 2 | 3 | 4 | 5 | 6 | 7 | 8 |
| --- | --- | --- | --- | --- | --- | --- | --- | --- | --- | --- |
| B | Argenta | 0 | | 0 | 0 | 0 | 7 | 13 | 0 | 0 |
| F | Arizona | 0 | | 20 | 0 | 0 | 0 | 0 | 0 | 0 |
| B | Banff | 5 | | 0 | 0 | 0 | 15 | 0 | 0 | 0 |
| A | Bowron Lake | 4 | | 0 | 0 | 0 | 0 | 0 | 11 | 5 |
| D | California | 0 | | 0 | 0 | 20 | 0 | 0 | 0 | 0 |
| B | Canmore | 3 | | 0 | 0 | 0 | 13 | 2 | 0 | 0 |
| B | Cascade | 13 | | 0 | 0 | 0 | 0 | 6 | 0 | 1 |
| A | Chilcotin | 0 | | 0 | 0 | 0 | 0 | 0 | 3 | 6 |
| E | Colorado | 0 | | 2 | 18 | 0 | 0 | 0 | 0 | 0 |
| B | Cranbrook | 1 | | 0 | 1 | 0 | 9 | 8 | 0 | 0 |
| B | Crowsnest | 1 | | 0 | 0 | 0 | 17 | 0 | 0 | 0 |
| B | Cypress Hills (AB) | 0 | | 0 | 0 | 0 | 6 | 5 | 0 | 0 |
| B | Cypress Hills (SK) | 0 | | 0 | 0 | 0 | 7 | 4 | 1 | 0 |
| A | Fairview | 1 | | 0 | 0 | 0 | 0 | 0 | 8 | 11 |
| B | Falkland | 8 | | 0 | 0 | 0 | 6 | 6 | 0 | 0 |
| A | Farwell Canyon | 6 | | 0 | 0 | 0 | 0 | 0 | 5 | 9 |
| A | Fox Creek | 0 | | 0 | 0 | 0 | 0 | 0 | 12 | 8 |
| A | Fraser Lake | 0 | | 0 | 0 | 0 | 0 | 0 | 9 | 11 |
| A | Fort St James | 0 | | 0 | 0 | 0 | 0 | 0 | 14 | 6 |
| A | Fort St John | 0 | | 0 | 0 | 0 | 0 | 0 | 5 | 6 |
| B | Gladstone | 8 | | 0 | 0 | 0 | 7 | 5 | 0 | 0 |
| B | Golden | 3 | | 0 | 0 | 0 | 13 | 2 | 0 | 1 |
| A | Grande Prairie | 0 | | 0 | 0 | 0 | 0 | 0 | 13 | 7 |
| A | Houston | 0 | | 0 | 0 | 0 | 0 | 0 | 3 | 8 |
| C | Idaho | 2 | | 0 | 0 | 1 | 12 | 5 | 0 | 0 |
| B | Kamloops | 11 | | 0 | 0 | 0 | 2 | 8 | 0 | 0 |
| B | Kelowna | 4 | | 0 | 0 | 0 | 5 | 2 | 0 | 0 |
| B | Kimberley | 5 | | 0 | 0 | 0 | 13 | 2 | 0 | 0 |
| B | Kootenay/Yoho | 5 | | 0 | 1 | 0 | 10 | 2 | 0 | 0 |
| B | Lac Le Hache | 8 | | 0 | 0 | 0 | 1 | 4 | 2 | 5 |
| B | Lake Louise | 3 | | 0 | 1 | 0 | 10 | 7 | 0 | 0 |
| A | Lesser Slave Lake | 0 | | 0 | 0 | 0 | 0 | 0 | 13 | 6 |
| B | Lillooet | 9 | | 0 | 0 | 1 | 1 | 4 | 1 | 4 |
| A | Mackenzie | 0 | | 0 | 0 | 0 | 0 | 0 | 12 | 8 |
| B | Manning Park | 6 | | 0 | 0 | 6 | 2 | 2 | 0 | 0 |
| A | McBride | 3 | | 0 | 0 | 0 | 1 | 1 | 3 | 12 |
| B | Merritt | 9 | | 0 | 0 | 0 | 4 | 7 | 0 | 0 |
| C | Montana | 0 | | 0 | 3 | 0 | 13 | 4 | 0 | 0 |
| B | Mt Robson | 13 | | 0 | 0 | 1 | 0 | 6 | 0 | 0 |
| D | Nevada | 0 | | 0 | 0 | 18 | 0 | 0 | 0 | 0 |
| D | Oregon | 0 | | 0 | 0 | 20 | 0 | 0 | 0 | 0 |
| A | Peace Area | 0 | | 0 | 0 | 0 | 0 | 0 | 12 | 8 |
| B | Peachland | 11 | | 0 | 0 | 0 | 3 | 6 | 0 | 0 |
| A | Prince George | 0 | | 0 | 0 | 0 | 0 | 0 | 1 | 4 |
| A | Quesnel | 1 | | 0 | 0 | 0 | 0 | 1 | 4 | 11 |
| A | Smithers | 0 | | 0 | 0 | 0 | 0 | 0 | 7 | 10 |
| F | South Dakota | 0 | | 20 | 0 | 0 | 0 | 0 | 0 | 0 |
| B | Sparwood | 2 | | 0 | 0 | 0 | 14 | 4 | 0 | 0 |
| A | Tatla Lake | 0 | | 0 | 0 | 0 | 0 | 0 | 6 | 13 |
| A | Telkwa | 0 | | 0 | 0 | 0 | 0 | 0 | 8 | 12 |
| A | Terrace | 0 | | 0 | 0 | 0 | 0 | 0 | 3 | 11 |
| A | Tumbler Ridge | 0 | | 0 | 0 | 0 | 0 | 0 | 9 | 11 |
| E | Utah | 0 | | 0 | 20 | 0 | 0 | 0 | 0 | 0 |
| B | Valemount | 8 | | 0 | 0 | 0 | 0 | 6 | 1 | 0 |
| B | Valhalla | 2 | | 0 | 0 | 0 | 6 | 5 | 0 | 0 |
| C | Washington | 4 | | 0 | 0 | 10 | 2 | 4 | 0 | 0 |
| B | Wells Grey | 10 | | 0 | 0 | 0 | 0 | 9 | 1 | 0 |
| B | West Arm Park | 4 | | 0 | 0 | 1 | 10 | 5 | 0 | 0 |
| B | Whistler | 5 | | 0 | 0 | 5 | 0 | 7 | 0 | 0 |
| A | Wilmore-Kakwa | 3 | | 0 | 0 | 0 | 2 | 2 | 6 | 3 |
| E | Wyoming | 0 | | 0 | 19 | 0 | 0 | 1 | 0 | 0 |
| B | Yoho | 2 | | 0 | 0 | 0 | 7 | 1 | 0 | 0 |

Table S4: Cluster assignment of individuals from DAPC analysis, Neutral Markers

| Region | Clusters | 1 | 2 | 3 | 4 | 5 | 6 | 7 | 8 |
| --- | --- | --- | --- | --- | --- | --- | --- | --- | --- |
| B | Argenta | 7 | 0 | 10 | 2 | 0 | 1 | 0 | 0 |
| F | Arizona | 0 | 0 | 0 | 0 | 0 | 0 | 0 | 20 |
| B | Banff | 11 | 0 | 9 | 0 | 0 | 0 | 0 | 0 |
| A | Bowron Lake | 0 | 8 | 0 | 4 | 8 | 0 | 0 | 0 |
| D | California | 0 | 0 | 0 | 0 | 0 | 0 | 20 | 0 |
| B | Canmore | 8 | 0 | 9 | 1 | 0 | 0 | 0 | 0 |
| B | Cascade | 3 | 1 | 1 | 14 | 0 | 0 | 1 | 0 |
| A | Chilcotin | 0 | 2 | 0 | 1 | 6 | 0 | 0 | 0 |
| E | Colorado | 0 | 0 | 0 | 0 | 0 | 18 | 1 | 1 |
| B | Cranbrook | 4 | 0 | 13 | 1 | 0 | 0 | 1 | 0 |
| B | Crowsnest | 3 | 0 | 15 | 0 | 0 | 0 | 0 | 0 |
| B | Cypress Hills (AB) | 3 | 0 | 7 | 0 | 0 | 1 | 0 | 0 |
| B | Cypress Hills (SK) | 6 | 1 | 3 | 0 | 0 | 2 | 0 | 0 |
| A | Fairview | 0 | 13 | 0 | 1 | 6 | 0 | 0 | 0 |
| B | Falkland | 9 | 0 | 7 | 4 | 0 | 0 | 0 | 0 |
| A | Farwell Canyon | 1 | 5 | 0 | 7 | 7 | 0 | 0 | 0 |
| A | Fox Creek | 0 | 12 | 0 | 0 | 8 | 0 | 0 | 0 |
| A | Fraser Lake | 0 | 10 | 0 | 0 | 10 | 0 | 0 | 0 |
| A | Fort St James | 0 | 15 | 0 | 0 | 5 | 0 | 0 | 0 |
| A | Fort St John | 0 | 8 | 0 | 0 | 3 | 0 | 0 | 0 |
| B | Gladstone | 8 | 0 | 6 | 4 | 0 | 2 | 0 | 0 |
| B | Golden | 5 | 0 | 12 | 2 | 0 | 0 | 0 | 0 |
| A | Grande Prairie | 0 | 10 | 0 | 0 | 10 | 0 | 0 | 0 |
| A | Houston | 0 | 3 | 0 | 1 | 7 | 0 | 0 | 0 |
| C | Idaho | 5 | 0 | 10 | 1 | 0 | 2 | 2 | 0 |
| B | Kamloops | 5 | 0 | 8 | 7 | 0 | 1 | 0 | 0 |
| B | Kelowna | 6 | 0 | 3 | 0 | 0 | 2 | 0 | 0 |
| B | Kimberley | 8 | 0 | 10 | 2 | 0 | 0 | 0 | 0 |
| B | Kootenay/Yoho | 7 | 0 | 9 | 1 | 0 | 1 | 0 | 0 |
| B | Lac Le Hache | 5 | 3 | 1 | 6 | 5 | 0 | 0 | 0 |
| B | Lake Louise | 12 | 0 | 6 | 0 | 0 | 3 | 0 | 0 |
| A | Lesser Slave Lake | 0 | 11 | 0 | 1 | 7 | 0 | 0 | 0 |
| B | Lillooet | 2 | 6 | 4 | 7 | 0 | 0 | 1 | 0 |
| A | Mackenzie | 0 | 14 | 0 | 0 | 6 | 0 | 0 | 0 |
| B | Manning Park | 1 | 0 | 5 | 5 | 0 | 0 | 5 | 0 |
| A | McBride | 1 | 7 | 0 | 8 | 3 | 1 | 0 | 0 |
| B | Merritt | 10 | 0 | 6 | 4 | 0 | 0 | 0 | 0 |
| C | Montana | 2 | 0 | 10 | 0 | 0 | 7 | 1 | 0 |
| B | Mt Robson | 4 | 0 | 6 | 9 | 0 | 0 | 1 | 0 |
| D | Nevada | 0 | 0 | 0 | 0 | 0 | 0 | 18 | 0 |
| D | Oregon | 0 | 0 | 0 | 0 | 0 | 0 | 20 | 0 |
| A | Peace Area | 0 | 13 | 0 | 0 | 7 | 0 | 0 | 0 |
| B | Peachland | 8 | 0 | 3 | 8 | 0 | 0 | 1 | 0 |
| A | Prince George | 0 | 2 | 0 | 0 | 3 | 0 | 0 | 0 |
| A | Quesnel | 1 | 5 | 0 | 3 | 8 | 0 | 0 | 0 |
| A | Smithers | 0 | 5 | 0 | 0 | 12 | 0 | 0 | 0 |
| F | South Dakota | 0 | 0 | 0 | 0 | 0 | 0 | 0 | 20 |
| B | Sparwood | 10 | 0 | 8 | 0 | 0 | 2 | 0 | 0 |
| A | Tatla Lake | 0 | 9 | 0 | 0 | 10 | 0 | 0 | 0 |
| A | Telkwa | 0 | 12 | 0 | 0 | 8 | 0 | 0 | 0 |
| A | Terrace | 0 | 3 | 0 | 0 | 11 | 0 | 0 | 0 |
| A | Tumbler Ridge | 0 | 9 | 0 | 0 | 11 | 0 | 0 | 0 |
| E | Utah | 0 | 0 | 0 | 0 | 0 | 20 | 0 | 0 |
| B | Valemount | 1 | 1 | 3 | 9 | 0 | 1 | 0 | 0 |
| B | Valhalla | 8 | 0 | 4 | 1 | 0 | 0 | 0 | 0 |
| C | Washington | 0 | 0 | 6 | 4 | 0 | 0 | 10 | 0 |
| B | Wells Grey | 6 | 2 | 8 | 3 | 0 | 1 | 0 | 0 |
| B | West Arm Park | 11 | 0 | 7 | 2 | 0 | 0 | 0 | 0 |
| B | Whistler | 0 | 0 | 1 | 10 | 0 | 1 | 5 | 0 |
| A | Wilmore-Kakwa | 2 | 6 | 1 | 4 | 3 | 0 | 0 | 0 |
| E | Wyoming | 0 | 0 | 0 | 0 | 0 | 20 | 0 | 0 |
| B | Yoho | 5 | 0 | 5 | 0 | 0 | 0 | 0 | 0 |

Table S5: Cluster assignment of individuals from DAPC analysis, Adaptive Markers

| Region | Clusters | 1 | 2 | 3 | 4 | 5 | 6 | 7 | 8 |
| --- | --- | --- | --- | --- | --- | --- | --- | --- | --- |
| B | Argenta | 5 | 1 | 8 | 1 | 2 | 0 | 0 | 3 |
| F | Arizona | 0 | 0 | 0 | 0 | 0 | 0 | 20 | 0 |
| B | Banff | 9 | 0 | 1 | 5 | 3 | 0 | 0 | 2 |
| A | Bowron Lake | 0 | 9 | 0 | 3 | 1 | 7 | 0 | 0 |
| D | California | 1 | 0 | 0 | 0 | 2 | 0 | 1 | 16 |
| B | Canmore | 9 | 0 | 4 | 2 | 2 | 0 | 0 | 1 |
| B | Cascade | 2 | 2 | 4 | 9 | 0 | 0 | 0 | 3 |
| A | Chilcotin | 0 | 4 | 1 | 0 | 0 | 4 | 0 | 0 |
| E | Colorado | 0 | 0 | 0 | 0 | 9 | 0 | 11 | 0 |
| B | Cranbrook | 6 | 0 | 4 | 2 | 6 | 0 | 0 | 1 |
| B | Crowsnest | 9 | 0 | 3 | 3 | 1 | 0 | 0 | 2 |
| B | Cypress Hills (AB) | 2 | 0 | 4 | 3 | 1 | 0 | 0 | 1 |
| B | Cypress Hills (SK) | 2 | 0 | 3 | 0 | 3 | 1 | 0 | 3 |
| A | Fairview | 0 | 8 | 0 | 2 | 0 | 10 | 0 | 0 |
| B | Falkland | 5 | 3 | 2 | 3 | 1 | 0 | 0 | 6 |
| A | Farwell Canyon | 1 | 8 | 1 | 7 | 0 | 3 | 0 | 0 |
| A | Fox Creek | 0 | 11 | 0 | 0 | 0 | 8 | 0 | 1 |
| A | Fraser Lake | 0 | 9 | 0 | 1 | 1 | 9 | 0 | 0 |
| A | Fort St James | 1 | 5 | 0 | 1 | 0 | 13 | 0 | 0 |
| A | Fort St John | 0 | 6 | 1 | 0 | 0 | 4 | 0 | 0 |
| B | Gladstone | 2 | 0 | 7 | 4 | 5 | 0 | 0 | 2 |
| B | Golden | 9 | 1 | 2 | 4 | 2 | 0 | 0 | 1 |
| A | Grande Prairie | 0 | 6 | 0 | 1 | 0 | 13 | 0 | 0 |
| A | Houston | 0 | 2 | 0 | 0 | 0 | 9 | 0 | 0 |
| C | Idaho | 5 | 0 | 5 | 1 | 2 | 0 | 1 | 6 |
| B | Kamloops | 3 | 2 | 8 | 5 | 0 | 0 | 0 | 3 |
| B | Kelowna | 2 | 0 | 3 | 4 | 0 | 0 | 0 | 2 |
| B | Kimberley | 7 | 0 | 6 | 5 | 1 | 0 | 0 | 1 |
| B | Kootenay/Yoho | 9 | 0 | 1 | 5 | 3 | 0 | 0 | 0 |
| B | Lac Le Hache | 2 | 7 | 2 | 8 | 0 | 1 | 0 | 0 |
| B | Lake Louise | 9 | 1 | 4 | 2 | 3 | 0 | 0 | 2 |
| A | Lesser Slave Lake | 0 | 6 | 1 | 1 | 0 | 11 | 0 | 0 |
| B | Lillooet | 3 | 8 | 3 | 5 | 0 | 0 | 0 | 1 |
| A | Mackenzie | 0 | 7 | 0 | 0 | 0 | 12 | 0 | 1 |
| B | Manning Park | 2 | 0 | 4 | 6 | 1 | 0 | 0 | 3 |
| A | McBride | 1 | 7 | 2 | 1 | 0 | 9 | 0 | 0 |
| B | Merritt | 6 | 2 | 2 | 4 | 1 | 1 | 0 | 4 |
| C | Montana | 4 | 1 | 4 | 3 | 5 | 0 | 0 | 3 |
| B | Mt Robson | 8 | 0 | 6 | 4 | 1 | 0 | 0 | 1 |
| D | Nevada | 0 | 0 | 0 | 0 | 0 | 0 | 0 | 18 |
| D | Oregon | 3 | 0 | 0 | 1 | 0 | 0 | 1 | 15 |
| A | Peace Area | 0 | 6 | 1 | 1 | 0 | 12 | 0 | 0 |
| B | Peachland | 5 | 1 | 5 | 5 | 1 | 0 | 0 | 3 |
| A | Prince George | 1 | 2 | 0 | 0 | 0 | 2 | 0 | 0 |
| A | Quesnel | 2 | 9 | 0 | 3 | 0 | 2 | 0 | 1 |
| A | Smithers | 1 | 4 | 0 | 1 | 0 | 11 | 0 | 0 |
| F | South Dakota | 0 | 0 | 0 | 0 | 1 | 0 | 19 | 0 |
| B | Sparwood | 9 | 0 | 5 | 1 | 3 | 0 | 0 | 2 |
| A | Tatla Lake | 0 | 8 | 0 | 3 | 0 | 8 | 0 | 0 |
| A | Telkwa | 0 | 8 | 0 | 0 | 0 | 12 | 0 | 0 |
| A | Terrace | 1 | 4 | 0 | 0 | 0 | 9 | 0 | 0 |
| A | Tumbler Ridge | 0 | 7 | 1 | 0 | 0 | 11 | 0 | 1 |
| E | Utah | 0 | 0 | 0 | 0 | 18 | 0 | 2 | 0 |
| B | Valemount | 1 | 2 | 5 | 3 | 0 | 4 | 0 | 0 |
| B | Valhalla | 4 | 0 | 4 | 2 | 3 | 0 | 0 | 0 |
| C | Washington | 5 | 0 | 4 | 7 | 1 | 0 | 0 | 3 |
| B | Wells Grey | 1 | 2 | 12 | 3 | 1 | 0 | 0 | 1 |
| B | West Arm Park | 7 | 0 | 5 | 3 | 3 | 0 | 0 | 2 |
| B | Whistler | 1 | 0 | 5 | 7 | 0 | 0 | 0 | 4 |
| A | Wilmore-Kakwa | 2 | 3 | 2 | 2 | 2 | 5 | 0 | 0 |
| E | Wyoming | 0 | 0 | 1 | 0 | 19 | 0 | 0 | 0 |
| B | Yoho | 5 | 0 | 1 | 3 | 1 | 0 | 0 | 0 |


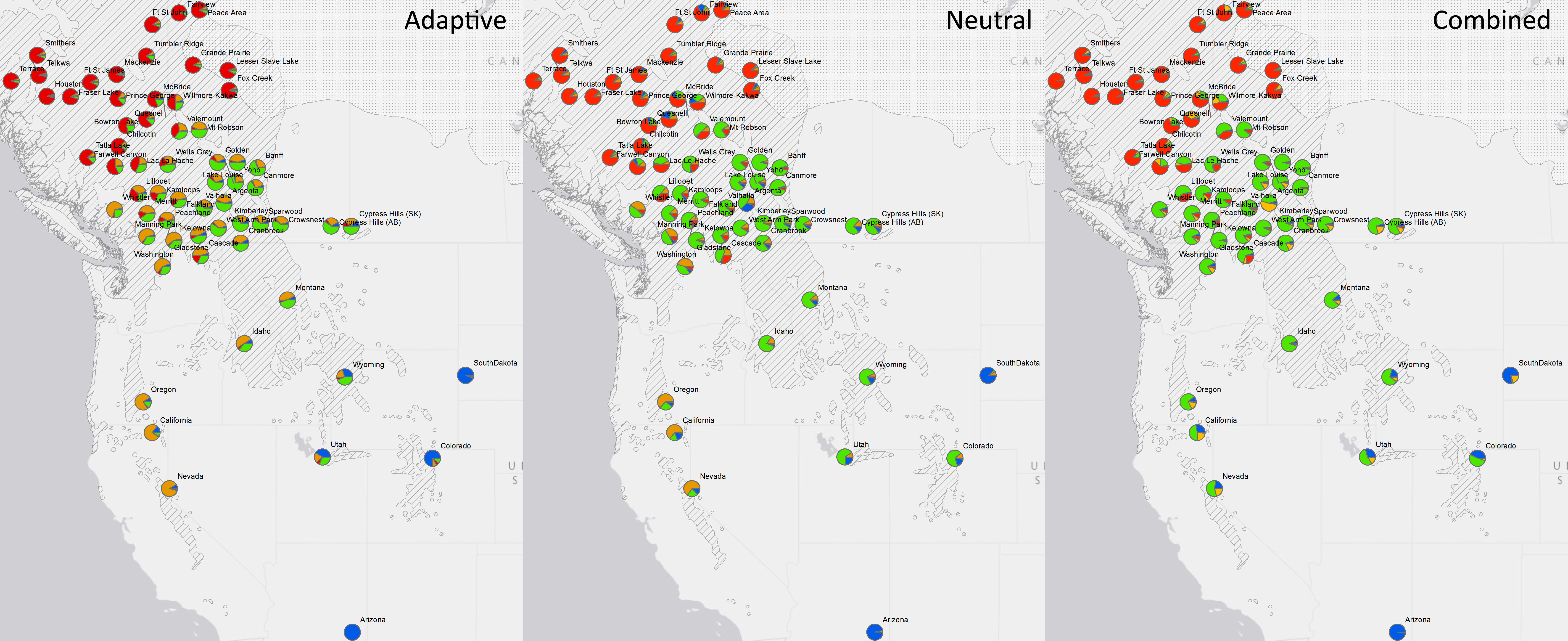


Figure S1. Cluster assignments from STRUCTURE for three sets of SNPs for K=4. The four general regions identified from the combined SNPs include northern Canada (red), southern Canada and Idaho, Montana and Washington (green), Oregon, California and Nevada (yellow), Utah and Wyoming Colorado, Arizona and South Dakota (blue).


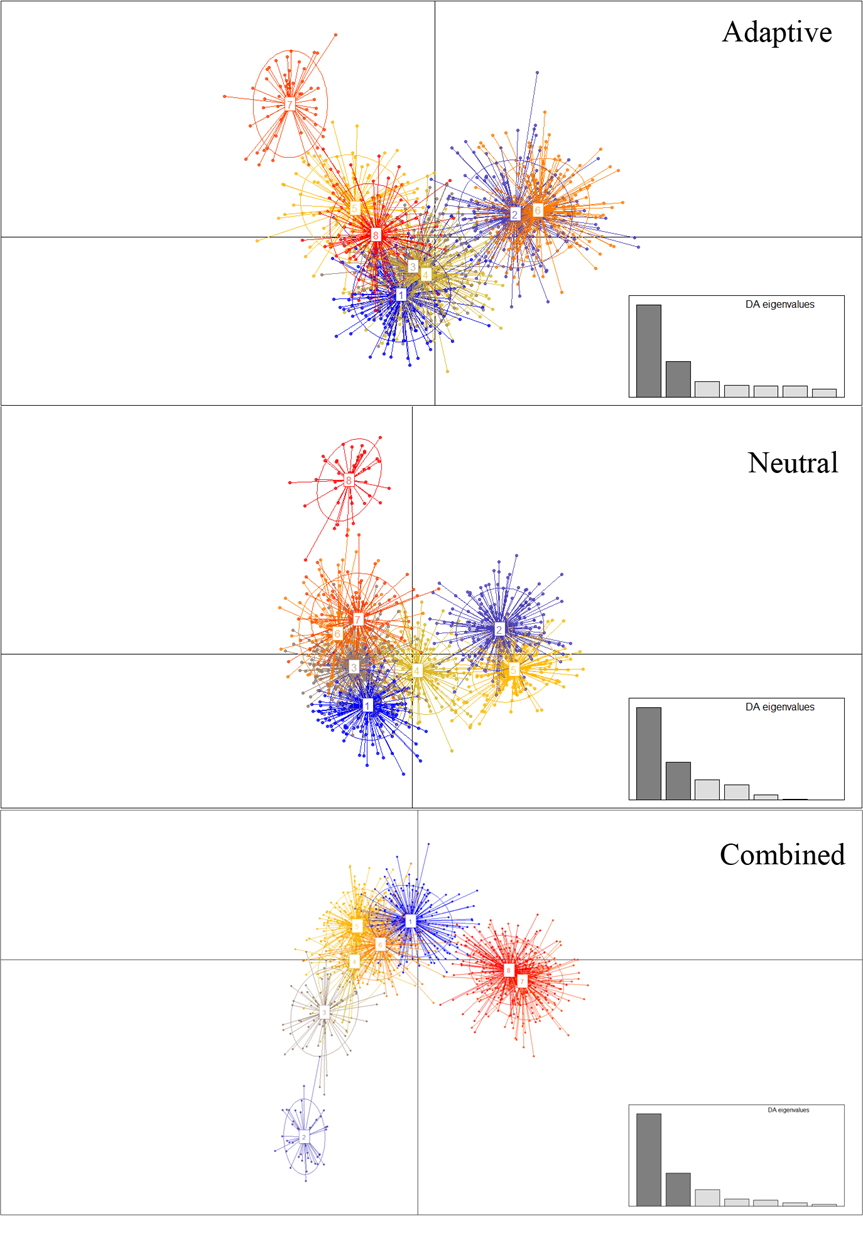


Figure S2. DAPC clustering of each marker set for K=8.
